# *Mycobacterium tuberculosis* and Human Immunodeficiency Virus Type 1 Cooperatively Modulate Macrophage Apoptosis via Toll Like Receptor 2 and Calcium Homeostasis

**DOI:** 10.1371/journal.pone.0131767

**Published:** 2015-07-01

**Authors:** Subhash Mehto, Cecil Antony, Nabab Khan, Rahul Arya, Arti Selvakumar, Brijendra K Tiwari, Mohit Vashishta, Yogendra Singh, Shahid Jameel, Krishnamurthy Natarajan

**Affiliations:** 1 Infectious Disease Immunology Lab, Dr. B. R. Ambedkar Centre for Biomedical Research, University of Delhi, Delhi 110007, India; 2 Virology Group, International Centre for Genetic Engineering and Biotechnology, Aruna Asaf Ali Marg, New Delhi 110067, India; 3 CSIR-Institute of Genomics and Integrative Biology, Mall Road, Delhi 110007, India; Vita-Salute San Raffaele University School of Medicine, ITALY

## Abstract

The emergence of drug resistant strains of *Mycobacterium tuberculosis* (*M*. *tuberculosis*) together with reports of co-infections with the human immunodeficiency virus (HIV) has renewed interest to better understand the intricate mechanisms prevalent during co-infections. In this study we report a synergistic effect of *M*. *tuberculosis* and HIV-1, and their antigens Rv3416 and Nef, respectively, in inhibiting apoptosis of macrophages. This inhibition involves the TLR2 pathway and second messengers that play complementing and contrasting roles in regulating apoptosis. Interestingly, the route of calcium influx into cells differentially regulates apoptosis during antigenic co-stimulation. While calcium released from intracellular stores was anti-apoptotic, calcium influx from the external milieu was pro-apoptotic. Further, molecular sensors of intracellular calcium release aid in antigen mediated inhibition of apoptosis. A cross-regulation between oxidative burst and differential routing of calcium influx governed apoptosis. Interestingly, the HIV-1 Nef supported anti-apoptotic responses in macrophages whereas Vpu had no significant effect. These results point to a synergistic liaison between *M*. *tuberculosis* and HIV-1 in regulating macrophage apoptosis.

## Introduction

*Mycobacterium tuberculosis* (*M*. *tuberculosis*) and the human immunodeficiency virus (HIV) are intracellular pathogens that cause tuberculosis (TB) and Acquired Immuno Deficiency Syndrome (AIDS), respectively. The risk of developing TB is estimated to be several times greater in people with an underlying HIV infection than among those without it. Despite the development of new drugs the global burden of TB remains enormous. In 2012, there were an estimated 8.6 million new cases of TB of which 13% were co-infected with HIV, 1.3 million people died from TB, including almost one million deaths among HIV-negative individuals and 320,000 among people who were HIV-positive [1].

With the emergence of drug resistant TB [2] there is an even greater need to understand host-pathogen interactions during co-infection for better disease management and the development of new therapeutic interventions. Co-infection with HIV and *M*. *tuberculosis* leads to alterations in the clinical course of both diseases [3]. For example, *M*. *tuberculosis*-specific CD4^+^ T cells of infected individuals have impaired IFN-γ secreting capacity despite having improved CD4^+^ T cell counts [4].

Inhibition of cell death for persistent survival inside the host cell is one of the strategies used by both HIV and *M*. *tuberculosis*. During co-infection *M*. *tuberculosis* and HIV express and secrete proteins/antigens that modulate the infected cell as well as the extracellular environment towards establishment of a successful and persistent infection [5, 6]. Since macrophages serve as long-term hosts for both these pathogens [7, 8], deciphering the mechanisms that modulate macrophage survival is important for understanding host-pathogen interactions.

To that end, we investigated the role of *M*. *tuberculosis* and HIV-1 and their antigens in regulating macrophage apoptosis. Our results point to a synergistic effect of *M*. *tuberculosis* and HIV-1 in promoting anti-apoptotic responses in macrophages using TLR2 and calcium homeostatic dependent mechanisms.

## Material and Methods

### Cell Culture and Differentiation

The monocyte-macrophage cell line THP1 and human PBMCs derived macrophages were used in this study. THP-1 cell line was a kind gift from Dr. Pawan Sharma from International Centre for Genetic Engineering and Biotechnology, New Delhi [9]. Cells were maintained in RPMI-1640 medium supplemented with 10% FBS and 2 mmol/L L-glutamine. THP1 cells were differentiated into macrophages by incubation with 50 ng/ml of PMA for 16 h. HEK293T cells and the HIV indicator cell line TZM-bl (NIH AIDS Reagent Bank, USA) were cultured in DMEM containing 10% serum. The following biopharmacological inhibitors were used against various molecules—IP_3_R, 3,4,5-trimethoxybenzoic acid 8-(diethylamino) octyl ester (TMB-8) (100 μM); calcium influx, EGTA (3 mM); iNOS, N-Nitro-L-arginine methyl ester hydrochloride (L-NAME) (50 μM). Additionally, where mentioned, cells were stimulated with 50 μM H_2_O_2_ for indicated time periods_._ Unless mentioned otherwise, cells were incubated with the above reagents for 1 h prior to stimulation with Rv3416 and Nef.

### Human Studies

All experiments were conducted following approval by the human ethics committee of Dr. B R Ambedkar Centre for Biomedical Research, University of Delhi. Following written informed consent, 5–10 ml venous blood from healthy volunteers was drawn and PBMCs were enriched as described below.

### Differentiation of human PBMCs derived macrophages

Human PBMCs were isolated from buffy coats from normal donors over a Histopaque-1077 gradient according to standard procedures [10]. Monocytes were purified from PBMCs by a 2 h adherence step at 37°C in complete medium. Non-adherent cells were washed off by extensive washing with DPBS without Ca^++^ or Mg^++^ and the remaining adherent cells (90% monocytes, as determined by flow cytometric analysis of forward scatter/side scatter, CD14 and CD11c staining) were immediately subjected to macrophage differentiation protocol. Briefly, 0.5-1x10^6^ cells/ml monocytes were re-suspended and cultured in RPMI 1640 supplemented with 10% autologous human serum, 25 mM HEPES, and 2 mM glutamine (complete medium) containing 50 ng/ml Macrophage-Colony Stimulating factor (M-CSF) for 5–7 days, with cytokine addition every second day, to obtain a homogenous population of macrophages. Differentiated macrophages were stimulated with Rv3416 or Nef or both for 24h. The cells were processed for FACS analyses or western blotted for various molecules as indicated below.

### Materials

Antibodies to Bax, Bid, cytochrome C, Bcl-2, pAKT1/2/3 (Ser 473)-R, AKT, Inhibitor of Apoptosis (IAP), Apoptosis Inducing Factor (AIF), β-actin, GAPDH, control and specific siRNAs against various genes, and Luminol kits for chemiluminescence detection were purchased from Santa-Cruz Biotechnologies (Santa Cruz, CA); TMB8 and EGTA, were purchased from Sigma Chemical Co. (St. Louis MA). Recombinant *M*.*tb* antigen Rv3416 was expressed and purified as described earlier [11, 12]. Recombinant Nef protein was expressed in *E*. *coli* Rosetta strain with a minimal hexahistidine tag and purified to apparent homogeneity on a Ni-NTA affinity matrix. Endotoxin levels were measured using the E-TOXATE LAL test (Sigma Chemical Co., St Louis MA) and were typically less than 0.03 EU/ml.

### Flow Cytometry

At the end of the incubation period, cells were stained with Annexin V-APC (eBiosciences, USA) according to the manufacturer’s protocol and acquired on FACS Calibur (Beckton & Dickinson). The data were analyzed using Cell Quest Pro software. No gates were applied for analyses.

### MTT assay

PMA treated THP-1 cells (1 × 10^4^ cells/well) were cultured in a 96-well plate at 37°C, and stimulated with Rv3416 (20 μg/ml) or Nef (15 μg/ml) or Rv3416 and Nef together with or without Pam_3_CSK_4_ for 24h. Cells were treated with EGTA, TMB-8 and H_2_O_2_, 1h prior to stimulation with antigen/protein. At the end of incubation period cells were thoroughly washed twice with 1XPBS, 20 μl of MTT solution (5 mg/ml in PBS) and 100 μl of medium was added to each well. After incubation for another 4h, the resultant formazan crystals were dissolved in dimethyl sulfoxide (100 μl) and the absorbance intensity measured by a microplate reader at 570 nm with a reference wavelength of 620 nm. All experiments were performed in triplet.

### Mitochondrial membrane potential assay

PMA treated THP-1 cells were stimulated with Rv316 or Nef and Rv3416 and Nef with or without Pam_3_CSK_4_ for 24h. After the incubation period cells were stained with 2 μM JC-1 in RPMI-1640 medium for another 30 min. At the end of incubation period cells were washed twice with 1XPBS and observed under confocal microscopy. Cells were visualized under green and red wavelengths.

### Confocal microscopy

Rv3416 and Nef were biotinylated using NHS biotin as per standard protocols. PMA treated THP1 cells were stimulated with PE-streptavidin-biotin conjugated Rv3416 (20 μg/ml) or PE-streptavidin-Biotin conjugated Nef (15 μg/ml) for different time intervals (30 min, 60 min, 120 min, 240 min, and 6 h). Z-stack images were collected at 1 μm intervals for internalization of proteins using confocal microscopy (Nikon C2).

### Transfection of THP1 cells with siRNA and stimulations

PMA treated THP1 cells (1 x 10^6^/ml) were transfected with 60 pmoles of siRNA against MyD88, TRAF-6, IRAK-1, STIM1, STIM2 and ORAI1 for 36 h using the Hiperfect transfection reagent (Qiagen) in OPTIMEM medium (Invitrogen). Five hours after transfection complete medium (RPMI-1640 supplemented with 10% serum) was added and the incubation continued for 36 h. Subsequently, cells were stimulated for various times and processed for FACS or western blotting as described below. Knockdown efficiency of various genes used in the study shown in (S1 Fig). Unless otherwise mentioned, cells were stimulated with the TLR2 ligand Pam_3_CSK_4_ at 1 μg/ml along with 20 μg/ml Rv3416 and/or 15 μg/ml Nef for the indicated times. At the end of the incubation period cells were either processed for FACS or western blotted for the indicated molecules.

### Infection of cells with *M*. *tuberculosis* H37Rv and stimulation with Nef

PMA treated THP1 cells (1 x 10^6^/ml) were stimulated with 1 μg/ml Pam3CSK4 and infected with *M*. *tuberculosis* H37Rv at 2 MOI with or without 15 μg/ml Nef and incubated for 24 h. At the end of the incubation period cells were either processed for FACS or western blotted for indicated molecules.

### Infection of cells with HIV-1 and stimulation with Rv3416

HEK cells (1 x 10^3^/ml) were transfected with 10 μg of the HIV-1 clade B infectious molecular clone plasmid pNL4-3 or its Vpu-deleted version pNL-∆Vpu or the Nef-deleted version pNL-∆Nef-IRES-GFP using the JetPrime reagent following standardized protocol. Thirty-six hours post-transfection supernatants were harvested and filtered through 0.4 μm filter (Millipore). The infectious virus was quantified by infecting the HIV indicator cell line TZM-bl followed by the ß-galactosidase assay 48 h post-infection. PMA stimulated THP-1 macrophages (1 x 10^6^/ml) were starved by culturing in serum-free RPMI for 2 h followed by infection with 0.5 MOI of wild type or mutant HIV-1 NL4-3 along with 1 μg/ml Pam3CSK4 with or without 20 μg/ml Rv3416. The cells were then incubated for 48 h. At the end of incubation period, cells were processed for FACS analyses.

### Western blotting for signaling molecules

At the end of incubation, cells were chilled on ice, washed once with ice cold PBS and lysed in buffer containing 10 mM HEPES (pH 7.9), 10 mM KCl, 0.1 mM EDTA, 0.1 M EGTA, 0.5% Nonidet P-40, and 2 μg/ml each of Aprotinin, Leupeptin and Pepstatin. The suspension was centrifuged at 13,000 x g for 5 min at 4°C. The supernatant containing 25 μg protein was resolved on 10% SDS-PAGE and subsequently transferred onto nitrocellulose membranes (Hybond C pure, Amersham Biosciences, Arlington Heights, IL). The blots were then probed with antibodies to various molecules, followed by HRP-labeled secondary antibodies. The blots were later developed by chemiluminescence using the Luminol reagent. A separate gel was run and probed for either β-actin or GAPDH as a loading control.

### Subcellular fractionation for mitochondrial proteins

PMA treated THP-1 cells were stimulated with Rv3416 or Nef or Rv3416 and Nef. After the incubation period cells were harvested at 500 g for 5 min at 4°C. The pellet was suspended in fractionation buffer A (10 mM HEPES pH 7.4, 0.1 mM EDTA, 1mM EGTA and 250 mM Sucrose along with protease inhibitors) and homogenized using 26 gauge needle for 5–6 times. Homogenized sample was then centrifuged twice at 700 g for 10 min at 4°C. The supernatant was collected and centrifuged at 7000 g for 20 min at 4°C. The supernatant was designated as the cytosolic fraction. The pellet containing the mitochondrial fraction was washed twice in fractionation buffer B (10 mM HEPES pH 7.4, 5 mM KH_2_PO_4,_ 5 mM succinate and 250 mM sucrose). The pellet was suspended in fractionation buffer B and incubated at -80°C overnight for lysis. Protein concentration was measured using Bio-Rad Bradford reagent. Cytosolic contamination in mitochondrial fraction was checked by Western blotting for GAPDH.

### Statistical Analyses

Two-tailed Student’s t test was carried out to test the significance of the results. A P value of less than 0.05 was taken as statistically significant difference.

## Results

### *M*. *tuberculosis* Rv3416 and HIV Nef antigens synergistically inhibit macrophage apoptosis

In order to investigate the role of *M*. *tuberculosis* and HIV antigens in modulating macrophage apoptosis, we used two antigens that are known to modulate macrophage defense functions. We previously identified a number of *M*. *tuberculosis* antigens that are expressed in infected macrophages as a function of time [11]. These antigens downmodulated immune responses to *M*. *tuberculosis* by attenuating pro-inflammatory cytokine expression, promoting Th2 responses and downregulating MHC class I and class II and cytokine receptors on macrophages. Amongst all the antigens expressed inside infected macrophages at 5 days post-infection, Rv3416 was the most potent in downregulating anti-bacterial responses. Further, Rv3416 is reported to promote latency in *M*. *tuberculosis* infected macrophages [11]. Further, we recently also showed the role of Rv3416 in downregulating immune responses from dendritic cells in a TLR2 and DC-SIGN specific mechanisms that involved specific genes in the calcium and cysteine protease pathways [12]. We reasoned that since in most cases, *M*. *tuberculosis* infection precedes HIV infection [13], Rv3416 would be an ideal choice to investigate modulation of macrophage functions in conjunction with HIV or its antigens. Similarly, Nef is the earliest protein to be expressed following HIV infection of macrophages or CD4^+^ T cells and has been widely studied for its effects on regulating the host cell environment for increased viral replication and also known to regulate apoptosis in macrophages [14]. We have recently shown the effects of Nef on miRNA-mediated innate restriction in a monocyte/macrophage experimental system [15, 16].

For this study we used THP-1 macrophages since they have been known to mimic functions of macrophages and monocytes [17]. Therefore, to begin with, we stimulated THP1 macrophages for 24 h and 48 h with Rv3416 and Nef either individually or together, and monitored the levels of various molecules that influence apoptosis. We first ensured that the two proteins are taken up by macrophages. To that end we biotinylated the two proteins and incubated them with streptavidin conjugated phycoerythrin (PE). PMA stimulated macrophages were then incubated with Rv3416 or Nef for different time points and analyzed by confocal microscopy. Z-stacking of the cells was carried out and data is presented in (S2 Fig). As shown in the figure both proteins were internalized in significant amounts to mediate the effects.

**Fig 1:**
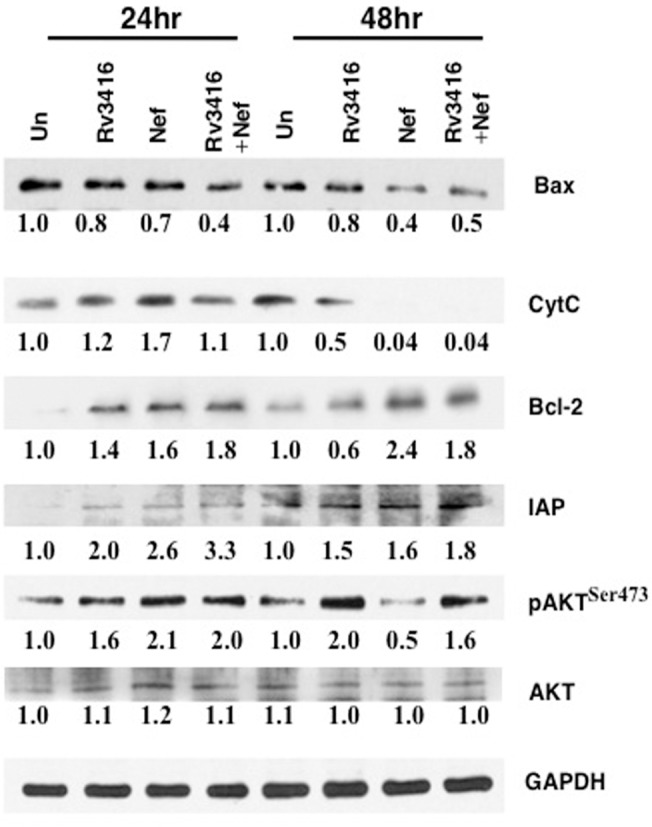
Rv3416 and Nef synergistically inhibit apoptosis in macrophages. PMA stimulated THP1 cells were stimulated with 20 μg/ml Rv3416 or 15 μg/ml Nef or both for 24h or 48h. Cytoplasmic extracts were probed for indicated molecules and analyzed by western blots. Numbers below the blots indicate the relative intensities of the bands. Data from one of three experiments are shown.

Next, we investigated the effects of these two antigens in mediating cell survival of macrophages. As shown in (Fig 1), both Rv3416 and Nef increased the expression levels of Bcl-2, pAkt and Inhibitor of Apoptosis (IAP), while decreasing the levels of Bax. For many molecules costimulation had a greater effect when compared to individual stimulations. Although a marginal increase of cytochrome C was observed following individual stimulation by the two antigens at 24h, co-stimulation decreased its levels at 24h and more significantly at 48h. This indicated that both antigens modulated macrophage apoptosis and when present together the effect was synergistic in nature. We further measured the cytosolic as well as mitochondrial levels of cytochrome C. As shown in (S3 Fig), at 48h, bulk of the cytochrome C levels was accumulated in mitochondria and not in cytosol indicating a plausible role for cytochrome C in modulating apoptosis upon costimulation of macrophages with the two antigens. We also monitored apoptosis by Annexin V staining along with propidium iodide staining in order to distinguish apoptotic responses from necrotic responses. Results depicted in (S4A Fig) clearly establish increased percentage of cells inhibiting apoptosis following stimulation with Rv3416 and Nef. We further monitored percentage survival by MTT assay (S4B Fig), wherein stimulation of cells with Rv3416 and Nef showed increased cell survival when compared with individual stimulation. In addition, we also monitored apoptosis by JC-1 staining of cells. JC-1 is a dye that stains mitochondria red when its membrane is intact and green when its membrane is depolarized and cells proceed through apoptosis [18]. As shown in (S4C Fig), costimulation with both Rv3416 and Nef increased red fluorescence of cells indicating inhibition of apoptosis when compared with either unstimulated cells or individual stimulations with either Rv3416 or Nef. These results once again indicated a synergistic role of Rv3416 and Nef in inhibiting apoptosis of macrophages.

### Costimulation of TLR2 with Rv3416 and Nef modulates macrophages survival

**Fig 2:**
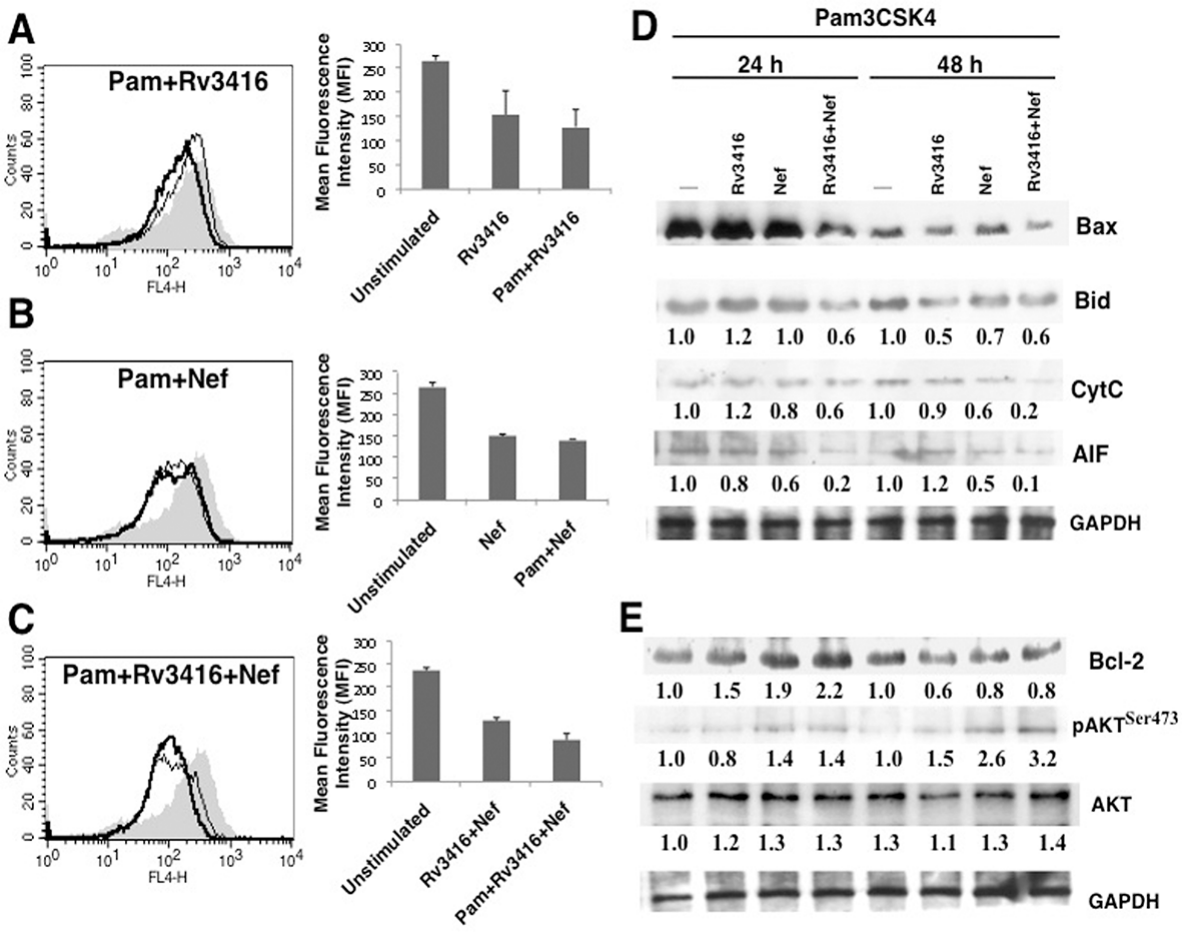
TLR2 co-stimulation exacerbates Rv3416 and Nef mediated inhibition of apoptosis in macrophages. PMA stimulated THP1 cells were stimulated with 20 μg/ml Rv3416 (Panel A, thin line) or 15 μg/ml Nef (Panel B, thin line) or both (Panel C, thin line) or in the presence of 1 μg/ml TLR2 ligand Pam3CSK4 (Panel A-C; indicated as Pam) for 24h. Cells were stained with Annexin V-APC. Thick lines in Panels (A-C) represent stimulation as indicated in the figure. Bar graphs adjacent to histograms in Panel (A-C) show relative MFIs of the histograms. Data from one of three independent experiments are shown. For Panel D and E, PMA stimulated THP1 cells were stimulated with 1 μg/ml Pam3CSK4 along with 20 μg/ml Rv3416 or 15 μg/ml Nef or both for 24h or 48h. Cytoplasmic extracts were prepared post-stimulation and western blotted for indicated molecules. Numbers below the blots indicate the relative intensities of the bands. Data from one of three experiments are shown. In Panel C, P<0.016 for Unstimulated vs Rv3416+Nef; P< 0.023 for Rv3416+Nef vs Pam+Rv3416+Nef.

Toll Like Receptor 2 (TLR2) plays a decisive role in mediating both protective and suppressive responses during *M*. *tuberculosis* and HIV infections [19–21]. To investigate the role of TLR2 in the above process, we stimulated macrophages with Pam3CSK4, a known TLR2 ligand, along with Rv3416 or Nef, and monitored apoptosis with Annexin V staining as well as the expression levels of pro- and anti-apoptotic molecules. Stimulation of macrophages with Pam3CSK4 alone had no significant effect on Annexin V staining over that of unstimulated control (S5 Fig). While stimulation of macrophages with either Rv3416 or Nef decreased Annexin V staining on macrophages (Fig 2A and 2B, thin lines), stimulation with Pam3CSK4 along with either Rv3416 (Fig 2A, thick line) or with Nef (Fig 2B, thick line) had no significant effect on Annexin V staining when compared in the absence of Pam3CSK4 stimulation. Interestingly, however, co-stimulation of macrophages with Pam3CSK4 along with Rv3416 and Nef (Fig 2C, thick line) decreased Annexin V staining when compared in the absence of TLR2 stimulation (Fig 2C, thin line). Bar charts alongside Panels A-C represent the Mean Fluorescence Intensities (MFIs) of the corresponding panels. The results in (Fig 2A–2C) indicated that TLR2 potentiated inhibition of apoptosis only upon co-stimulation with the two antigens and not upon individual stimulation with either antigen.

We next monitored the levels of key pro-apoptotic and anti-apoptotic molecules in the context of TLR2 and antigenic stimulations. As shown in (Fig 2D), stimulation of TLR2 in combination with Rv3416 and Nef decreased the levels of pro-apoptotic molecules Bax, Bid, cytochrome C (Cyt C) and Apoptosis Inducing Factor (AIF). In contrast to pro-apoptotic molecules, the levels of anti-apoptotic molecules Bcl2 and pAkt were significantly increased upon co-stimulation with the two antigens along with TLR2 stimulation (Fig 2E). These results reaffirm the anti-apoptotic roles of the two antigens in a TLR2 dependent pathway. Likewise, we monitored apoptosis by Annexin V-propidium iodide staining (S6A Fig), percent cell survival by MTT assay (S6B Fig) and JC-1 staining (S6C Fig) when stimulated with either Rv3416 or Nef or both in the context of TLR2 stimulation. The results were in agreement with the data presented in (Fig 2).

We further extended the observations obtained with THP-1 macrophages to human blood monocyte derived macrophages. As shown in (S7 Fig), stimulation of blood macrophages with either Rv3416 or Nef decreased Annexin V staining and costimulation with Rv3416 and Nef further decreased Annexin V staining. These observations indicate that like in THP-1 cells, *M*.*tb* and HIV antigens inhibited apoptosis in primary blood macrophages also.

To investigate whether the observed effects were specific to TLR2, we evaluated the role of different TLRs in regulating macrophage survival following co-stimulation with Rv3416 and Nef. To that end we stimulated TLR4, TLR7, TLR9 and DC-SIGN (another receptor for *M*. *tuberculosis* and HIV) [22–24] with their specific ligands–lipopolysaccharide (LPS), Imiquimod, CpG DNA and mannosylated lipoarabinomanan (manLAM), respectively. As shown in (S8A Fig) and in contrast to TLR2, no significant modulations in Annexin V levels were observed with TLR4, TLR7 or TLR9. A marginal increase in Annexin V levels was observed upon stimulation of DC-SIGN upon stimulation with Nef but not with Rv3416. However, no significant changes were observed when cells were costimulated with Rv3416 and Nef. These results indicate that both antigens preferentially employ the TLR2 pathway in inducing anti-apoptotic responses in macrophages. Similar to results obtained on Annexin V staining, stimulation of cells with ligands to TLR4 or TLR7 or TLR9 or DC-SIGN in the context of the two antigens did not induce any significant changes in the expression levels of either AIF or Bcl2 (S8B Fig). These results indicated a minimal role for these pattern recognition receptors in mediating macrophage survival.

**Fig 3:**
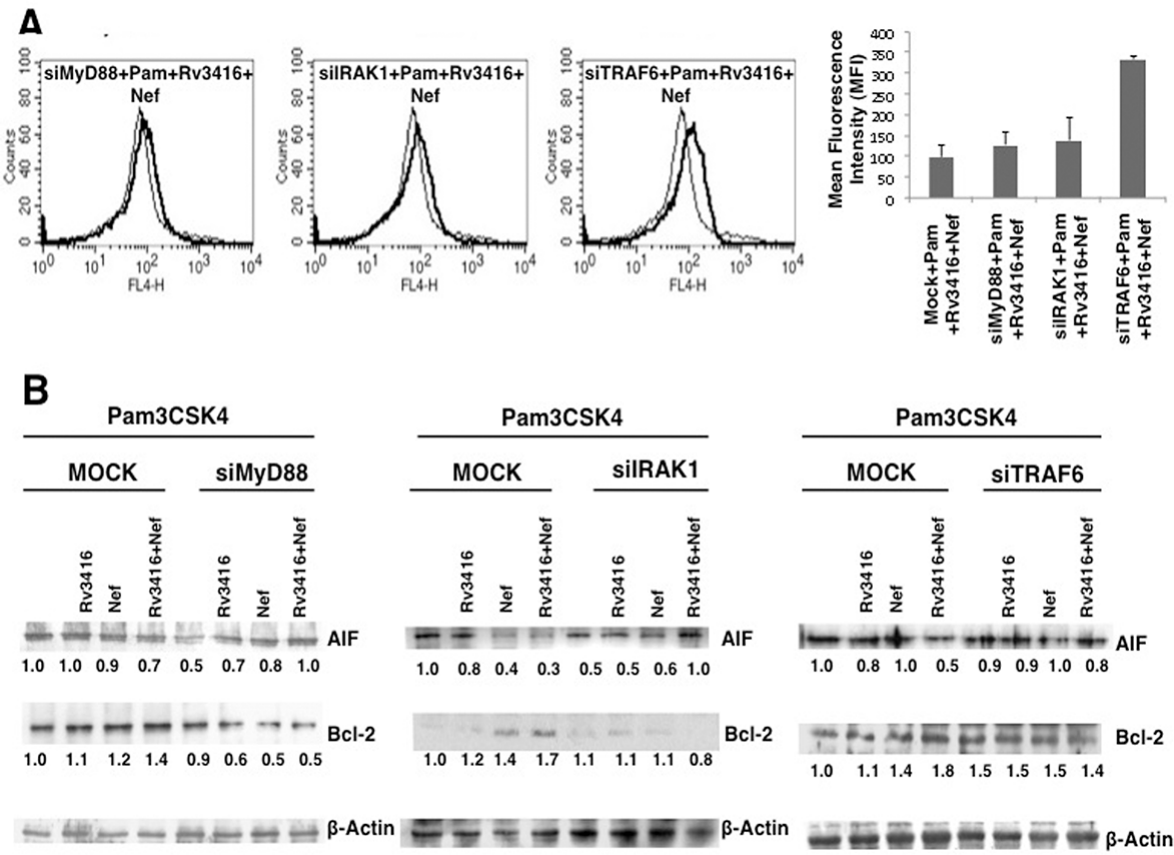
MyD88, IRAK1 and TRAF6 regulate Rv3416 and Nef mediated inhibition of apoptosis in macrophages. For Panel A, PMA stimulated THP1 cells were transfected with control siRNAs (thin lines) or specific siRNAs to indicated molecules (thick lines) for 36h followed by stimulations with 1 μg/ml Pam3CSK4 (Pam) along with 20 μg/ml Rv3416 and 15 μg/ml Nef for 24h. Cells were stained with Annexin V-APC. Data from one of three independent experiments are shown. Bar graphs adjacent to histograms in Panel A show relative MFIs of the histograms. For Panel B, cytoplasmic extracts from cells cultured as described above were probed for indicated molecules and analyzed by western blots. MOCK represents cells transfected with control siRNAs. Numbers below the blots indicate the relative intensities of the bands. Data from one of three experiments are shown. In Panel A, P<0.04 Control siRNA+Pam+Rv3416+Nef vs siTRAF6+Pam+Rv3416+Nef.

Since HIV-1 Nef is known to recruit downstream molecules of TLR pathways such as TRAF6, TRAF5 and TRAF2 and modulate HIV-1 replication in macrophage [25], therefore, to further confirm the role of TLR2 in mediating macrophage apoptosis, we next investigated the role of different intermediates in the TLR signaling pathway. To that end using specific siRNAs we individually knockdown MyD88, TRAF6 and IRAK1 and monitored apoptosis by Annexin V staining and the expression levels of AIF and Bcl2 as representative pro- and anti-apoptotic molecules, respectively. As shown in (Fig 3A and 3B), Annexin V staining was increased upon knockdown of MyD88 (albeit marginally), IRAK1 or TRAF6. Similarly, co-stimulation with Rv3416 and Nef along with TLR2 stimulation increased the levels of AIF and marginally decreased the levels of Bcl2 following knockdown of IRAK1. These results indicate that the two antigens employ the TLR pathway in regulating macrophage apoptosis during HIV/*M*. *tuberculosis* co-infection.

### Calcium homeostasis regulates macrophage apoptosis by Rv3416 and Nef

Calcium is an important second messenger that governs a number of cellular processes [26]. Calcium concentrations in the cells also influence cell survival by directly regulating apoptosis [27]. Our earlier work has indicated a dominant role for Voltage Gated Calcium Channels (VGCC) in suppressing macrophage functions during *M*. *tuberculosis* infection [28]. Inhibiting VGCC upregulated pro-inflammatory cytokines and induced Th1 responses and activated macrophages to kill intracellular *M*. *tuberculosis*. Further, patients with active TB expressed high levels of L-type VGCC that was attenuated following chemotherapy, and inhibiting L-type VGCC attenuated *M*. *tuberculosis* infection in mice [28].

**Fig 4:**
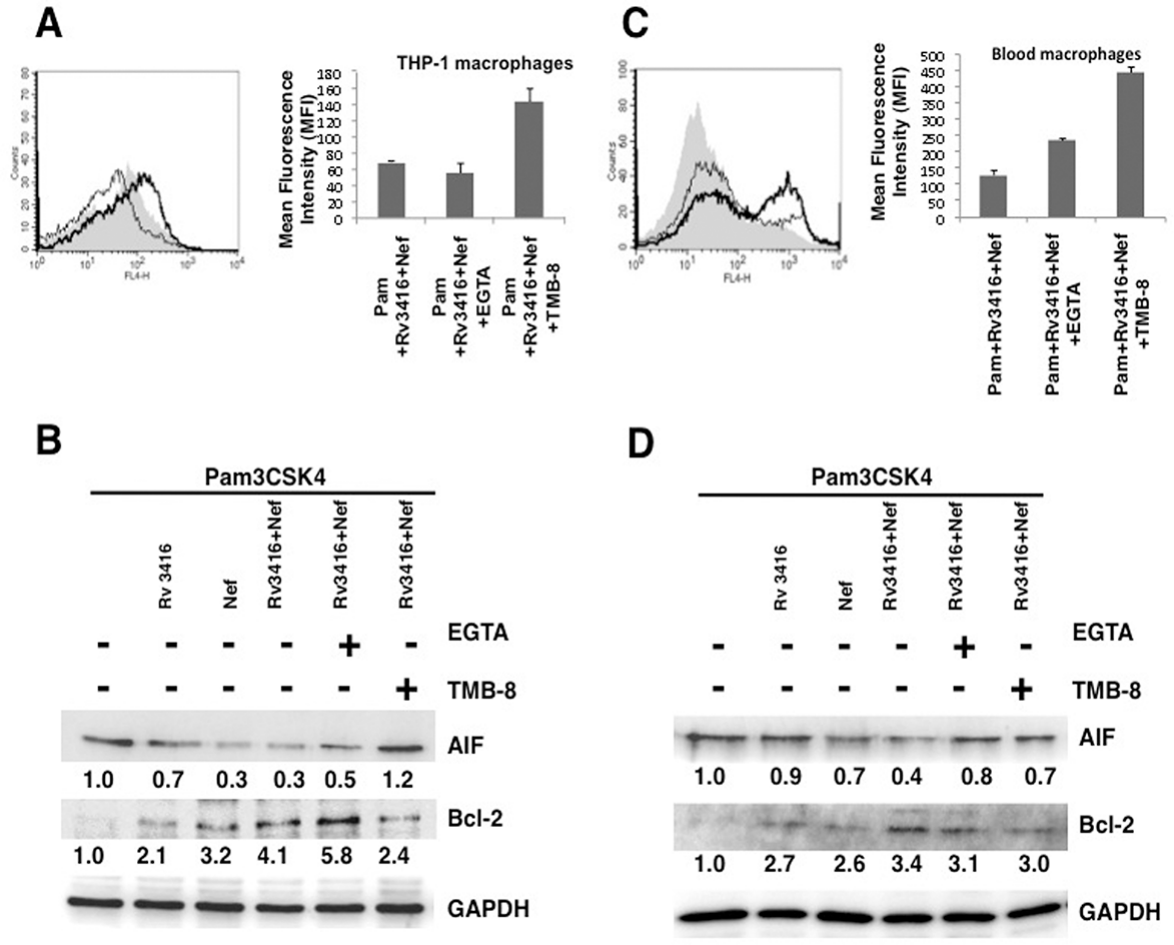
Route of calcium influx differentially regulates apoptosis in macrophages by Rv3416 and Nef. Either PMA stimulated THP1 macrophages (Panel A and B) or blood monocyte derived macrophages (Panel C and D) were treated with inhibitor to either calcium influx from external medium (EGTA; thin lines) or calcium influx from internal stores (TMB-8; thick lines) for 1h and stimulated with 1 μg/ml Pam3CSK4 along with 20 μg/ml Rv3416 and 15 μg/ml Nef for 24h. At the end of the incubation period cells were stained with Annexin V-APC. Shaded histograms in Panel A and Panel C represent cells stimulated with 1 μg/ml Pam3CSK4 along with 20 μg/ml Rv3416 and 15 μg/ml Nef for 24h. Bar graphs adjacent to histograms in Panel A and Panel C show relative MFIs of the histograms. Data from one of three independent experiments are shown. For Panel B (THP-1 macrophages) and Panel D (blood macrophages) were treated as indicated for 24h. Cytoplasmic extracts were probed for indicated molecules and analyzed by western blots. Numbers below the blots indicate the relative intensities of the bands. Data from one of three experiments are shown. In Panel A, P<0.04 for Pam+Rv3416+Nef vs Pam+Rv3416+Nef+EGTA. P<0.01 for Pam+Rv3416+Nef vs Pam+Rv3416+Nef+TMB8. In Panel C, P<0.05 for Pam+Rv3416+Nef vs Pam+Rv3416+Nef+EGTA. P<0.03 for Pam+Rv3416+Nef vs Pam+Rv3416+Nef+TMB8.

Therefore, we next explored the role of calcium influx from internal stores and external medium in regulating Rv3416 and Nef mediated apoptosis. To that end, macrophages were treated with EGTA to chelate extracellular calcium or TMB-8 to inhibit store operated calcium release from IP_3_R. As shown in (Fig 4A and 4B and S9 Fig), calcium homeostasis from the external medium and the internal stores had contrasting effects. While inhibiting calcium influx from external medium (Fig 4A, thin line) complemented Rv3416 and Nef mediated inhibition of apoptosis, inhibiting store operated calcium release from the endoplasmic reticulum (Fig 4A, thick line) promoted apoptosis. This was also evident at the level of the relative expression levels of Bcl2 and AIF (Fig 4B). This indicated that the route of calcium influx had determinant effects on macrophage survival. While routing calcium influx from external medium was pro-apoptotic, calcium influx from internal stores was anti-apoptotic. Interestingly, co-inhibiting calcium from both external medium and internal stores together was anti-apoptotic suggesting a dominant effect of calcium release from internal stores over influx from extracellular milieu (data not shown). These results further point to a tight control of apoptotic machinery by different calcium routes.

We further extended these observations to human blood monocyte derived macrophages. Interestingly, inhibiting calcium influx from either external medium (Fig 4C, thin line) or calcium release from intracellular stores (Fig 4C, thick line) increased Annexin V staining and similarly increased the expression levels of AIF and decreased Bcl2 levels (Fig 4D). Despite differences in the mechanisms involved in regulating apoptosis in THP-1 and blood macrophages, these results, nevertheless pointed towards determinant role of calcium in modulating apoptosis during *M*. *tuberculosis* and HIV co-infections.

**Fig 5:**
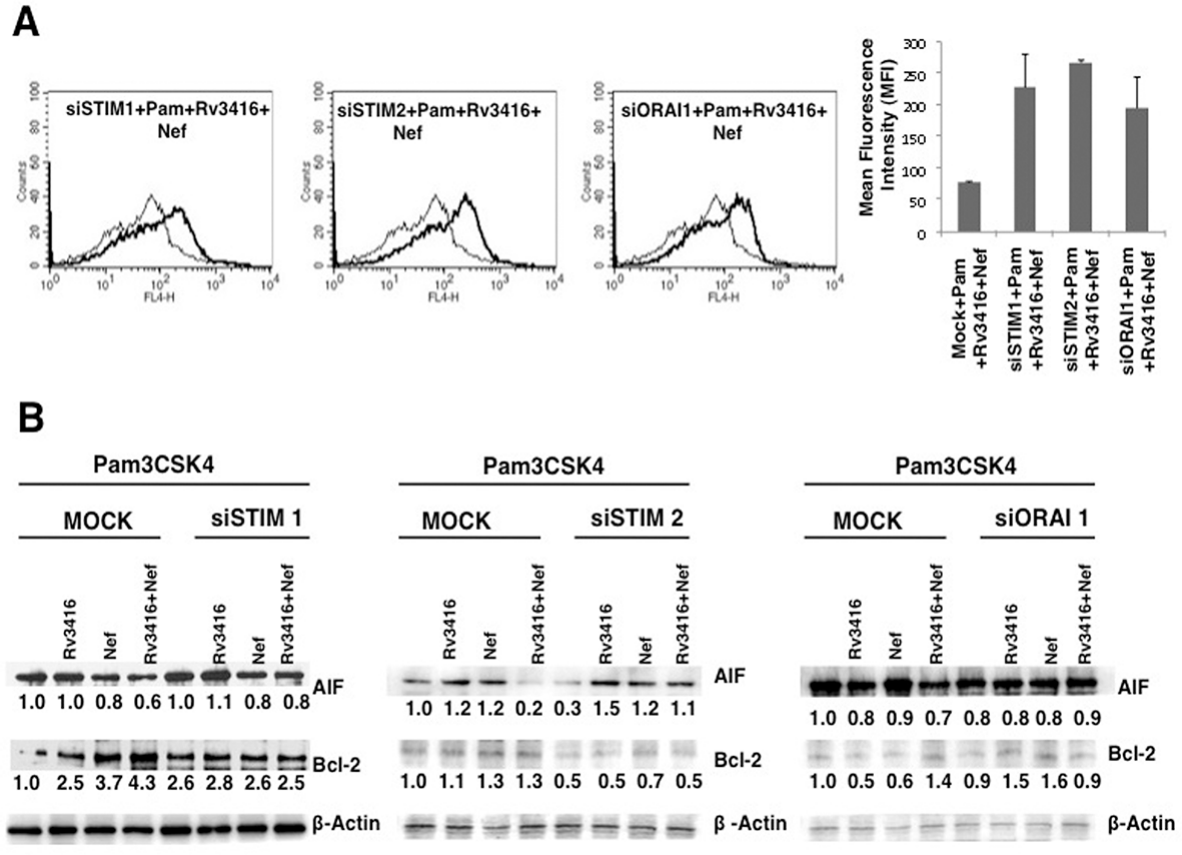
Molecular sensors of calcium influx regulate inhibition of apoptosis by Rv3416 and Nef in macrophages. For Panel A, PMA stimulated THP1 cells were transfected with control siRNAs (thin lines) or specific siRNAs to indicated molecules (thick lines) for 36h followed by stimulations with 1 μg/ml Pam3CSK4 (Pam) along with 20 μg/ml Rv3416 and 15 μg/ml Nef for 24h. Cells were stained with Annexin V-APC. Bar graphs adjacent to histograms in Panel A show relative MFIs of the histograms. Data from one of three independent experiments are shown. For Panel B, PMA stimulated THP1 cells were transfected with siRNAs to indicated molecules for 36h followed by stimulations with 1 μg/ml Pam3CSK4 along with 20 μg/ml Rv3416 or 15 μg/ml Nef or both for 24h. Cytoplasmic extracts were probed for indicated molecules and analyzed by western blots. MOCK represents cells transfected with control siRNAs. Numbers below the blots indicate the relative intensities of the bands. Data from one of three experiments are shown. In Panel A, P<0.009 for Pam+Rv3416+Nef vs Pam+Rv3416+Nef+siSTIM1; P<0.02 for Pam+Rv3416+Nef vs Pam+Rv3416+Nef+siSTIM2; P<0.01 for Pam+Rv3416+Nef vs Pam+Rv3416+Nef+siORAI1.

We further dissected the role of intracellular sensors regulating calcium homeostasis in governing apoptosis by the two antigens. We investigated the roles of STIM1 and STIM2, the molecular sensors that regulate Store Operated Calcium Entry (SOCE) via the Calcium Release Activated Calcium Channel (CRAC) ORAI1. As shown in (Fig 5), knockdown of STIM1, STIM2 or ORAI1, increased apoptosis by increasing Annexin V levels (Fig 5A) and levels of AIF while decreasing the levels of anti-apoptotic molecule Bcl2 (Fig 5B). This indicated that members of the SOCE pathway regulate macrophage apoptosis during *M*. *tuberculosis* and HIV co-infection. Further it also complemented the data in (Fig 4), wherein inhibiting calcium from internal stores was pro-apoptotic. This further established a critical role for calcium in regulating macrophage apoptosis during co-infection.

### Oxidative burst regulates macrophage apoptosis by Rv3416 and Nef

**Fig 6:**
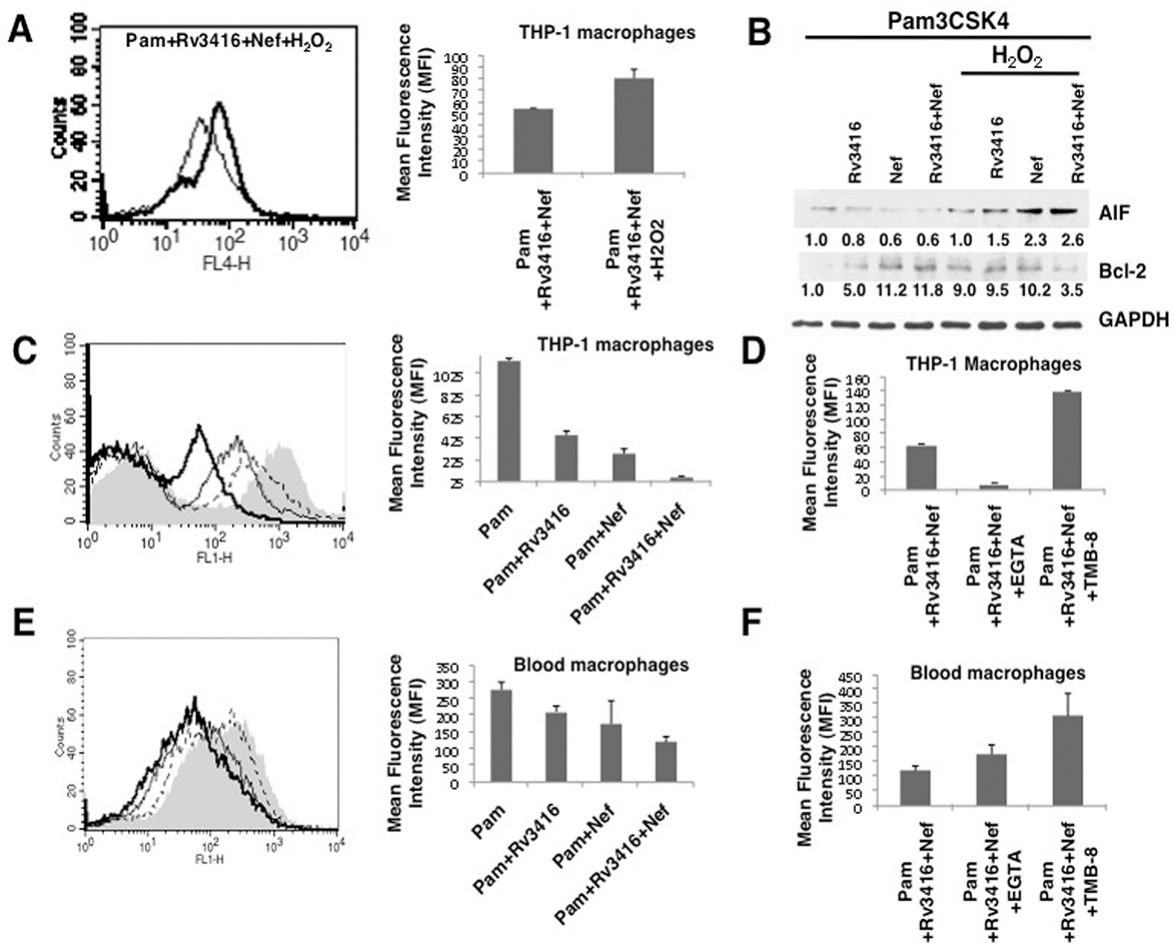
Reciprocal regulation of oxidative burst and calcium routing regulate apoptosis in macrophages by Rv3416 and Nef. For Panel A and B, PMA stimulated THP1 cells were treated with 50 μM H_2_O_2_ for 1h and stimulated with 1 μg/ml Pam3CSK4 along with 20 μg/ml Rv3416 and 15 μg/ml Nef for 24h. For Panel A, cells were stained with Annexin V-APC. Thin lines represent cells stimulated in the absence of H_2_O_2_ while the thick lines represent cells stimulated in the presence of H_2_O_2_. Data from one of three independent experiments are shown. Bar graphs adjacent to histogram in Panel A show relative MFIs of the histogram. For Panel B, PMA stimulated THP1 cells were stimulated with 1 μg/ml Pam3CSK4 along with 20 μg/ml Rv3416 or 15 μg/ml Nef or both for 24h and cytoplasmic extracts were probed for indicated molecules and analyzed by western blots. Numbers below the blots indicate the relative intensities of the bands. Data from one of three experiments are shown. For Panel C (PMA stimulated THP1 macrophages) and Panel E (Blood macrophages) were stimulated with 1 μg/ml Pam3CSK4 along with 20 μg/ml Rv3416 (dotted lines) or 15 μg/ml Nef (thin lines) or both (thick lines) for 2h for measuring oxidative burst. Thirty minutes prior to the incubation period, cells were loaded with 10μM DCFH-DA. At the end of the incubation period, cells were thoroughly washed with the culture medium and immediately analyzed for ROS levels by flow cytometry. Shaded histograms in Panel C and E represent cells stimulated with 1 μg/ml Pam3CSK4 only. Bar graphs adjacent to histograms in Panel C and Panel D show relative MFIs of the corresponding histograms. For Panel D (THP-1 macrophages) and Panel F (Blood macrophages) cells stimulated with 1 μg/ml Pam3CSK4 along with 20 μg/ml Rv3416 and 15 μg/ml Nef with or without EGTA or TMB-8 for 1h and ROS levels were measured. In Panel C P<0.004 for Pam vs Pam+Rv3416+Nef; In Panel D, P<0.005 for Pam+Rv3416+Nef vs Pam+Rv3416+Nef+TMB; P<0.005 for Pam+Rv3416+Nef vs Pam+Rv3416+Nef+EGTA. In Panel E, P<0.03 for Pam vs Pam+Rv3416+Nef; In Panel F, P<0.05 for Pam+Rv3416+Nef vs Pam+Rv3416+Nef+TMB; P<0.04 for Pam+Rv3416+Nef vs Pam+Rv3416+Nef+EGTA.

The generation of oxidative burst and nitric oxide is a key defense mechanism mounted by macrophages in response to infections by *M*. *tuberculosis* and HIV [29, 30]. In turn, reactive oxygen species (ROS) also regulate a number of cellular processes [31]. Therefore, we next investigated the role of ROS in regulating apoptosis by Rv3416 and Nef. As shown in (Fig 6A), enhancing ROS with H_2_O_2_ treatment negated the anti-apoptotic effect of the two antigens. This was true for both Annexin V staining (Fig 6A) as well as in the levels of AIF and Bcl2, wherein supplementing ROS increased AIF levels while reducing Bcl2 levels (Fig 6B). Bar graph adjacent to the (Fig 6A) shows relative MFIs of the histogram. However, inhibiting inducible nitric oxide had no significant effects (S10 Fig). This indicated that the inhibition of apoptosis was dependent on the ROS pathway when compared with the nitric oxide pathway.

We had earlier reported a cross-regulation of ROS and calcium during *M*. *tuberculosis* infection [32]. Supplementing ROS enhanced calcium influx while supplementing calcium enhanced ROS in dendritic cells infected with mycobacteria. To further dissect the cross-regulation of ROS and calcium we measured ROS following inhibition of calcium influx from the external medium and intracellular stores in the context of costimulation with Rv3416 and Nef. Stimulation of TLR2 (Fig 6C, shaded histogram) in the presence of Rv3416 (Fig 6C, dotted line) or Nef (Fig 6C, thin line) inhibited ROS levels that were further reduced following co-stimulation with both antigens (Fig 6C, thick line)). Bar graph adjacent to the (Fig 6C) shows relative MFIs of the histogram. Interestingly, inhibiting calcium influx from internal stores completely reversed the effects of costimulation with Rv3416 and Nef and regenerated ROS levels, but inhibiting calcium influx from the external medium further attenuated ROS generation (Fig 6D). These results were in agreement with the data in (Fig 4), wherein routes of calcium influx inversely regulated apoptosis by Rv3416 and Nef. Further, they also point towards an interesting reciprocal regulation of ROS and calcium that has a bearing on macrophage apoptosis by Rv3416 and Nef.

We further extended these results to human blood monocyte derived macrophages. As shown in (Fig 6E), stimulation with either Rv3416 (Fig 6E, dotted line) or Nef (Fig 6E, thin line) decreased ROS levels when compared with stimulation with TLR2 (Fig 6E, shaded histogram). Costimulation with both Rv3416 and Nef (Fig 6E, thick line) further decreased ROS levels. Bar graph adjacent to (Fig 6E) shows relative MFIs of the histogram. Interestingly, inhibiting calcium influx from external medium marginally increased ROS levels while inhibiting calcium release from internal stores significantly enhanced ROS levels following costimulation with TLR2, Rv3416 and Nef (Fig 6F). The collective results shown in (Fig 6) point towards complementary roles of calcium homeostasis and ROS in regulating apoptosis during *M*. *tuberculosis* and HIV co-infection.

### *M*. *tuberculosis* and HIV infection inhibits macrophage apoptosis

**Fig 7:**
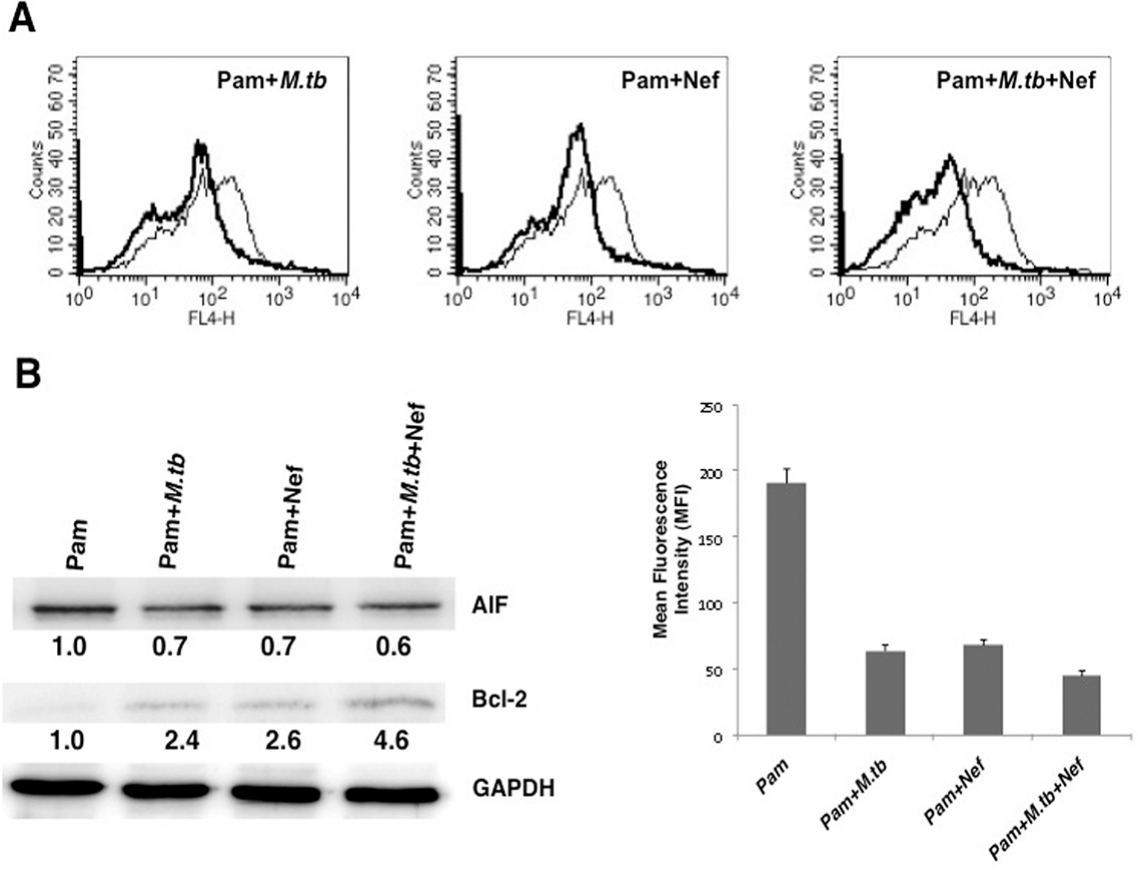
Nef synergizes with live *M*. *tuberculosis* infection to inhibit apoptosis in macrophages. PMA stimulated THP1 cells were stimulated with 1 μg/ml Pam3CSK4 (Pam) and infected with 2 MOI of *M*. *tuberculosis* (*M*.*tb*) H37Rv or stimulated with 15 μg/ml Nef or both for 24h. For Panel A, cells were stained with Annexin V-APC. Thin lines represent stimulation with Pam3CSK4 (Pam) alone and the thick lines represent stimulations as indicated. Bar graphs show relative MFIs of the histograms. Data from one of three independent experiments are shown. For Panel B, PMA stimulated THP1 cells were stimulated as indicated for 24h and cytoplasmic extracts were probed for indicated molecules and analyzed by western blots. Numbers below the blots indicate the relative intensities of the bands. Data from one of three experiments are shown. In Panel A, P<0.02 for Pam vs Pam+*M*.*tb*. P<0.02 for Pam vs Pam+Nef, P<0.006 for Pam vs Pam+*M*.*tb*+Nef.

We next investigated whether the effects observed with the antigens would be replicated during live infections. Since the containment requirements for *M*. *tuberculosis* and HIV are different, we could not co-infect macrophages with live HIV and live *M*. *tuberculosis*. Nevertheless, as a proof of concept we infected macrophages with either live virulent *M*. *tuberculosis* together with Nef stimulation or with live HIV infection together with Rv3416 stimulation. Infection of macrophages with virulent *M*. *tuberculosis* decreased Annexin V levels (Fig 7A) along with a concomitant decrease in AIF levels and an increase in Bcl2 levels (Fig 7B). Infection of macrophages with *M*. *tuberculosis* in the presence of Nef further inhibited apoptosis with a significant decrease in Annexin V levels (Fig 7A) and a sharp increase in Bcl2. However, there was no apparent decrease in AIF levels upon costimulation with *M*. *tuberculosis* and Nef over that observed with individual stimulations (Fig 7B). Nevertheless, these results clearly indicated the anti-apoptotic role of Nef in *M*. *tuberculosis* infections in macrophages.

**Fig 8:**
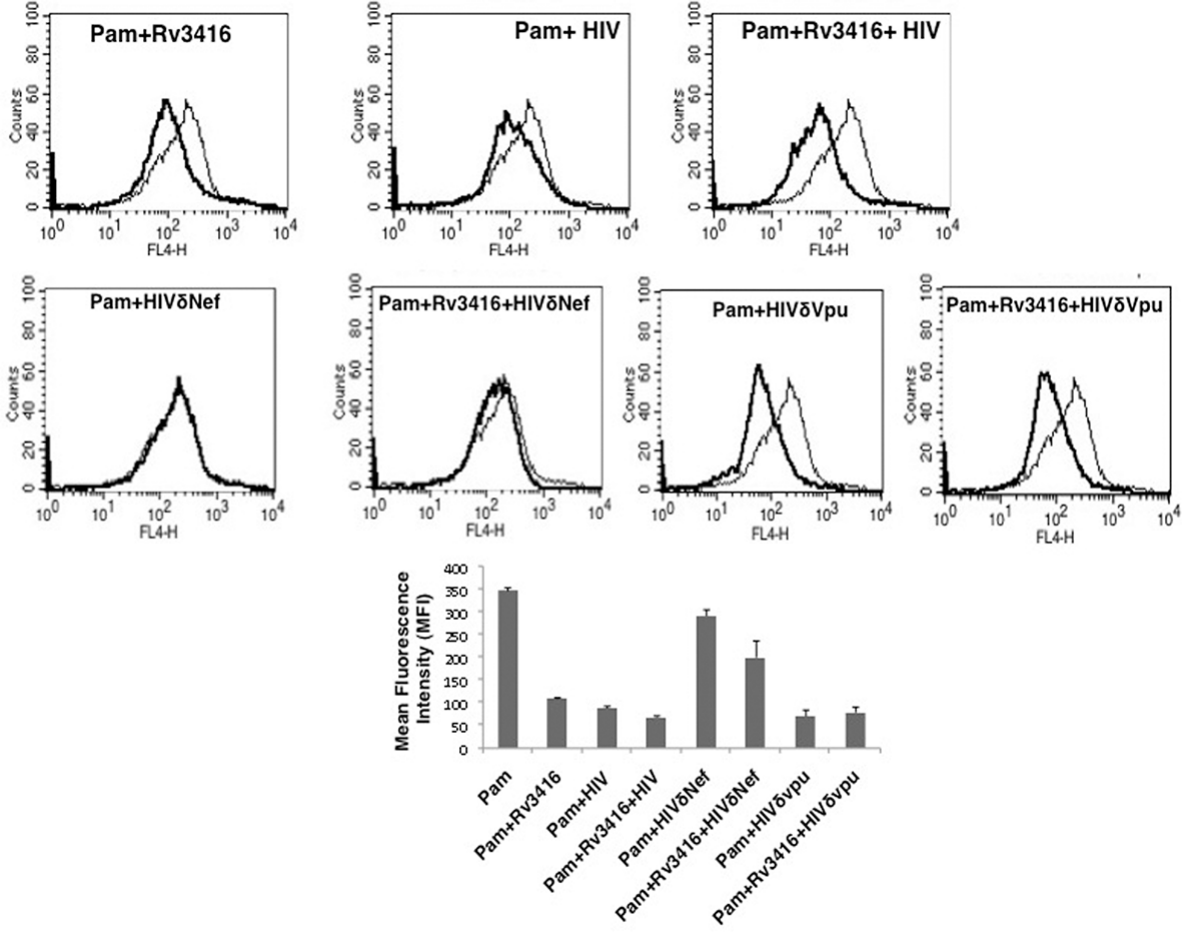
Rv3416 synergizes with Nef sufficient HIV but not Nef deficient HIV to inhibit apoptosis in macrophages. PMA stimulated THP1 cells were stimulated with 1 μg/ml Pam3CSK4 (Pam) and infected with 0.5 MOI of wild type HIV or Nef deficient HIV (HIVδNef) or Vpu deficient HIV (HIVδVpu) in the presence or absence of 20 μg/ml Rv3416 for 48h. Cells were stained with Annexin V-APC. Thin lines represent stimulation with Pam3CSK4 alone and the thick lines represent stimulation as indicated. Data from one of three independent experiments are shown. Bar graphs show relative MFIs of the histograms. Data from one of three experiments are shown. P<0.03 for Pam vs Pam+Rv3416. P<0.04 for Pam vs Pam+HIV, P<0.03for Pam vs Pam+Rv3416+HIV.

In the next set of experiments we infected macrophages with live HIV in the presence of Rv3416. Analogous to infection with *M*. *tuberculosis*, infection with HIV also inhibited apoptosis and Rv3416 treatment synergized with HIV to further inhibit macrophage apoptosis (Fig 8). These results confirm that co-infection with *M*. *tuberculosis* and HIV inhibits apoptosis in a synergistic manner.

To further establish the role of Nef in mediating anti-apoptotic responses during *M*. *tuberculosis* infections we infected macrophages with HIV incapable of expressing the Nef protein (HIVδNef). As shown in (Fig 8), infection with HIVδNef did not result in any reduction of Annexin V staining either in the absence or in the presence of Rv3416. In contrast to infection with HIVδNef, infection with HIVδVpu inhibited apoptosis to levels observed upon wild type HIV infection. Co-stimulation with HIVδVpu and Rv3416 also reduced Annexin V staining that was marginally more than that observed with HIVδVpu alone (Fig 8). These results clearly established a critical role for Nef in inhibiting apoptosis in macrophages.

## Discussion

Co-infections with *M*. *tuberculosis* and HIV pose a challenge towards developing therapeutic interventions to combat the mortality and morbidity associated with these pathogens. Therefore, a detailed understanding of the mechanisms that these two pathogens use to subvert host defense is critical. Although a number of studies have been carried out towards unraveling host-pathogen interactions, the role of specific antigens/proteins expressed by these two pathogens on modulating macrophage functions during costimulation or coinfections has not been investigated. In an effort to identify the pathways active during subversion of macrophage defense by these two pathogens and their antigens, this study focused on the regulation of apoptosis, which is a critical host response to limit the spread of intracellular pathogens. To counter this innate response, many pathogens inhibit apoptosis of infected cells to successfully establish infection. While this is true for both *M*. *tuberculosis* [33] and HIV [34], data on the cooperation, if any, between the two pathogens towards modulation of the apoptotic machinery and pathways involved therein are lacking.

To that end, we chose to work with two antigens, namely Rv3416 and Nef and investigated their roles in modulating macrophage apoptosis. Rv3416 is an antigen expressed by *M*. *tuberculosis* that promotes latency in macrophages and was shown previously by us to mediate immune suppression [11, 12]. Similarly, HIV-Nef is an antigen that is expressed early during infection and functions to enhance viral replication through multiple mechanisms, including the modulation of apoptosis. To begin with we monitored the expression levels of key proteins in the apoptotic pathway following stimulation of macrophages with the two antigens either individually or in combination. Our results show that the two antigens, when added together, synergistically reduced apoptosis of macrophages by inhibiting the levels of pro-apoptotic molecules while concomitantly increasing the expression levels of anti-apoptotic molecules. Further, it is plausible that the effects of these two proteins could be in trans. However, the fact that the two proteins could be present in the same macrophages cannot be ascertained at the moment nor can it be ruled out.

Since the involvement of innate receptors is a key step in the modulation of macrophage defense functions, we next investigated the role of TLRs in the above responses. Our results pointed to a specific role of TLR2 in mediating the anti-apoptotic effects of the two antigens. This was true both at the level of Annexin V staining as well as with the expression levels of anti-apoptotic and pro-apoptotic molecules. Interestingly, TLR2 involvement was observed only when macrophages were co-stimulated with the two antigens and not upon individual stimulations. We also confirmed the role of signaling intermediates in the TLR pathway wherein knockdown of key molecules reversed the anti-apoptotic effects of the two antigens. Several studies have reported utilization of the TLR pathway by *M*. *tuberculosis* antigens. For example, the 19 kDa lipoprotein of *M*. *tuberculosis* uses the TLR2 pathway to inhibit IFN- mediated responses [20] as well as the activation of MHC class II (*CIITA*) promoter [35]. Likewise, HIV gp120 induces pro-inflammatory responses in genital epithelial cells in a TLR2 and TLR4 dependent manner [36]. Further, a recent report also showed a role for TLR2 in modulating HIV replication in CD4^+^ central memory T cells [21]. Additionally, TLR7 and TLR8 differentially modulate cytokine responses in different subsets of dendritic cells [37]. In light of the above reports our data provide insights into the role of TLR2 in mediating early anti-apoptotic responses during co-stimulation by *M*. *tuberculosis* and HIV antigens.

Our previous work identified crucial roles for calcium in regulating immune responses and survival of *M*. *tuberculosis* in dendritic cells and macrophages [12]. Therefore, we also investigated the role of this important second messenger during anti-apoptotic responses by the two antigens. The results point towards an interesting dual role for calcium that regulates both pro- and anti-apoptotic responses which depends on its routing inside the cells. While calcium from intracellular stores played an anti-apoptotic role, calcium influx from the extracellular milieu was pro-apoptotic. Though intracellular calcium generally plays a pro-apoptotic role [38], our study suggests unique roles for the routes of calcium entry in differential regulation of apoptosis by the two antigens. Furthermore, we also identified the role for SOCE calcium channels during co-infection by the two pathogens. To the best of our knowledge this is the first report on the role of SOCE calcium channels and the differential routing of calcium influx in regulating macrophage apoptosis by the two pathogens in a TLR2 dependent manner.

Building on this, we next investigated the role of reactive oxygen intermediates, which constitute a major defense response in macrophages, and its cross-regulation by calcium in mediating apoptosis by the two antigens. Our previous results showed that Rv3416 downmodulates oxidative burst in dendritic cells [11]. Interestingly, it has been reported that Nef modulates oxidative burst in a biphasic manner, causing a sharp initial increase followed by a delayed strong decrease in monocytes [39]. Our data showed that supplementing ROS in macrophages reversed the anti-apoptotic effects of Rv3416 and Nef. Furthermore, both antigens downmodulated TLR2 induced ROS levels in a synergistic manner. These data indicate a critical role for oxidative burst that cooperates with quantitative and qualitative routing of calcium influx. This interaction has a determinant effect on modulation of apoptosis in macrophages by *M*. *tuberculosis* and HIV.

Finally we replicated key experiments using live *M*. *tuberculosis* and HIV infections, and the results complement the data obtained with the antigens alone. Using deletion mutants we also unequivocally established a role for Nef in mediating anti-apoptotic responses in *M*. *tuberculosis* infected macrophages. Overall the above results point to an interesting interplay of signaling modules that are employed by the two pathogens to increase macrophage survival so as to create an immune privileged site and niche for persistent infection.

## Supporting Information

S1 Fig Knockdown efficiency of siRNAs to various molecules. PMA stimulated THP1 cells were transfected with siRNAs to indicated molecules for 36h. Cytoplasmic extracts were prepared and western blotted for indicated molecules. MOCK represents cells transfected with control siRNAs.
(DOCX)

S2 Fig Rv3416 and Nef are internalized by macrophages. PMA stimulated THP1 cells were incubated with PE-streptavidin-biotin conjugated Rv3416 (20μg/ml) or PE-streptavidin-biotin conjugated Nef (15μg/ml). Internalization of the two proteins was monitored using confocal microscopy. Images show Z-stacks of 1μm section for Rv3416 at 30min post-stimulation (Panel A) and for Nef at 60min post-stimulation (Panel B).
(DOCX)

S3 Fig Cytochrome C is localized in mitochondria. PMA stimulated THP1 cells were stimulated with 20 μg/ml Rv3416 or 15 μg/ml Nef or both. After 48 h cytoplasmic and mitochondrial extracts were prepared and western blotted for Cytochrome C levels. One of two independent experiments is shown.
(DOCX)

S4 Fig Rv3416 and Nef modulate apoptosis and not necrosis of macrophages. PMA stimulated THP1 cells were stimulated with either 20 μg/ml Rv3416 or 15μg/ml Nef or both for 24h. Panel A, Cells were stained with Annexin V-APC and propidium Iodide (PI) and analyzed by flow cytometry. The percentage of cells positive for PI or/and Annexin V-APC are indicated inside the quadrants. Data from one of three independent experiments are shown. Panel B, shows percentage cell viability as determined by MTT assay. Unstimulated cells were taken as 100% viable and cell viability was calculated as percentage of unstimulated cell. Each experiment was performed in triplicate (n = 3). For Panel C, cells were stimulated with 20 μg/ml Rv3416 or 15μg/ml Nef or both and incubated for 24h. Cells were further incubated with 2 μM JC-1 dye for 30 min and analyzed by confocal microscopy. Green color indicate the loss of mitochondrial membrane potential while red color indicate the intact mitochondrial membrane potential.
(DOCX)

S5 Fig Stimulation of macrophages with TLR2 ligand Pam3CSK4 does not modulate apoptosis. PMA stimulated THP1 cells were stimulated with 1 μg/ml TLR2 ligand Pam3CSK4 for 24h and stained with Annexin V-APC. Thin line represents unstimulated cells while the dotted line represents stimulation with Pam3CSK4.
(DOCX)

S6 Fig Rv3416 and Nef modulate apoptosis and not necrosis of macrophages in the context of TLR2. PMA stimulated THP1 cells were stimulated with 20 μg/ml Rv3416 or 15μg/ml Nef or both with 1 μg/ml TLR2 ligand Pam3CSK4 and incubated for 24h. Panel A, Cells were stained with Annexin V-APC and propidium Iodide (PI) and analyzed by flow cytometry. The percentage of cells positive for PI or/and Annexin V-APC are indicated inside the quadrants. Data from one of three independent experiments are shown. Panel B, shows percentage cell viability as determined by MTT assay. Pam3CSK4 (Pam) treated cells were taken as 100% viable and cell viability was calculated as percentage of Pam3CSK4 (Pam) treated cell. Each experiment was performed in triplicate (n = 3). For Panel C, PMA stimulated THP1 cells were stimulated with 20 μg/ml Rv3416 or 15μg/ml Nef or both with 1 μg/ml TLR2 ligand Pam3CSK4 and incubated for 24h. Cells were further incubated with 2 μM JC-1 dye for 30 min and analyzed by confocal microscopy. Green color indicate the loss of mitochondrial membrane potential while red color indicate the intact mitochondrial membrane potential.
(DOCX)

S7 Fig Rv3416 and Nef synergistically inhibit blood monocyte derived macrophages. Peripheral blood mononuclear cells were enriched from whole blood and monocytes were differentiated into macrophage as described in Materials and methods. Macrophages were stimulated with stimulated with 1 μg/ml TLR2 ligand Pam3CSK4 (Pam) and either 20 μg/ml Rv3416 or 15μg/ml Nef or both for 24h and stained with Annexin V-APC and analyzed by flow cytometry. Thin line represents the cells stimulated with 1 μg/ml TLR2 ligand Pam3CSK4 (Pam). Thick line represents cells stimulated with as indicated in the histogram. Data from one of three independent experiments are shown.
(DOCX)

S8 Fig Inhibition of apoptosis by Rv3416 and Nef is not mediated via TLR4 or TLR7 or TLR9 or DC-SIGN. PMA stimulated THP1 cells were stimulated with 20 μg/ml Rv3416 or 15 μg/ml Nef or both for 24h along with ligands to TLR4 (0.1 μg/ml LPS) or TLR7 (1.0 μg/ml Imiquimod) or TLR9 (2.0 μg/ml CpG DNA) or DC-SIGN (0.5 μg/ml mannosylated Lipoarabinomanan; manLAM). For Panel A, cells were stained with Annexin V-APC. Thin lines represent stimulation with respective TLR ligands alone while the thick lines represent stimulation with respective TLR ligands with or without indicated antigens. For Panel B, cytoplasmic extracts from cells stimulated as above were western blotted for indicated molecules. Numbers below the blots indicate the relative intensities of the bands.
(DOCX)

S9 Fig Calcium homeostasis differentially modulates Rv3416 and Nef induced apoptosis of macrophages. PMA stimulated THP1 cells were stimulated with 1 μg/ml TLR2 ligand Pam3CSK4 and along with 20 μg/ml Rv3416 and 15μg/ml Nef or EGTA or TMB-8 for 24h. Panel A, Cells were stained with Annexin V-APC and Propidium Iodide (PI) and analyzed by flow cytometry. The percentage of cells positive for PI or/and Annexin V-APC are indicated inside the quadrants. Data from one of three independent experiments are shown. Panel B, shows percentage cell viability as determined by MTT assay. Pam+Rv3416+Nef stimulated cells were taken as 100% viable and cell viability was calculated as percentage of Pam+Rv3416+Nef stimulated cell. Each experiment was performed in triplicate (n = 3).
(DOCX)

S10 Fig Apoptosis in macrophages by Rv3416 and Nef does not involve inducible nitric oxide synthase. PMA stimulated THP1 cells were treated with 50 μM L-NAME for 1h followed by stimulations with 1 μg/ml Pam3CSK4 along with 20 μg/ml Rv3416 and 15 μg/ml Nef for 24h. For Panel A, cells were stained with Annexin V-APC. Thin line represent cells stimulated in the absence of H_2_O_2_ while the thick line represent cells stimulated in the presence of L-NAME. Data from one of three independent experiments are shown. For Panel B, PMA stimulated cells were stimulated as indicated for 24h and cytoplasmic extracts were probed for indicated molecules and analyzed by western blots. Numbers below the blots indicate the relative intensities of the bands. Data from one of three experiments are shown.
(DOCX)
