# Supplementary material for: Mycobacterium tuberculosis and Human Immunodeficiency Virus Type 1 Cooperatively Modulate Macrophage Apoptosis via Toll Like Receptor 2 and Calcium Homeostasis
Source: PLoS One. 2015 Jul 1;10(7):e0131767. doi: 10.1371/journal.pone.0131767 (PMC4489497; doi:10.1371/journal.pone.0131767)
Supplement: S1 Fig — PMA stimulated THP1 cells were transfected with siRNAs to indicated molecules for 36h. Cytoplasmic extracts were prepared and western blotted for indicated molecules. MOCK represents cells transfected with control siRNAs. (DOCX) [file pone.0131767.s001.docx]

**
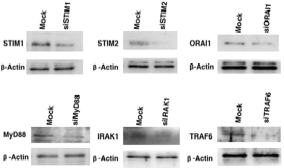
**

**S1 Fig. Knockdown efficiency of siRNAs to various molecules.** PMA stimulated THP1 cells were transfected with siRNAs to indicated molecules for 36h. Cytoplasmic extracts were prepared and western blotted for indicated molecules. MOCK represents cells transfected with control siRNAs.
