# Supplementary material for: Mycobacterium tuberculosis and Human Immunodeficiency Virus Type 1 Cooperatively Modulate Macrophage Apoptosis via Toll Like Receptor 2 and Calcium Homeostasis
Source: PLoS One. 2015 Jul 1;10(7):e0131767. doi: 10.1371/journal.pone.0131767 (PMC4489497; doi:10.1371/journal.pone.0131767)
Supplement: S2 Fig — PMA stimulated THP1 cells were incubated with PE-streptavidin-biotin conjugated Rv3416 (20μg/ml) or PE-streptavidin-biotin conjugated Nef (15μg/ml). Internalization of the two proteins was monitored using confocal microscopy. Images show Z-stacks of 1μm section for Rv3416 at 30min post-stimulation (Panel A) and for Nef at 60min post-stimulation (Panel B). (DOCX) [file pone.0131767.s002.docx]

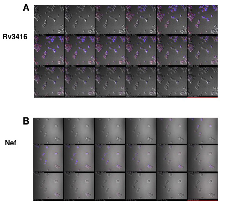


**S2 Fig. Rv3416 and Nef are internalized by macrophages.** PMA stimulated THP1 cells were incubated with PE-streptavidin-biotin conjugated Rv3416 (20μg/ml) or PE-streptavidin-biotin conjugated Nef (15μg/ml). Internalization of the two proteins was monitored using confocal microscopy. Images show Z-stacks of 1μm section for Rv3416 at 30min post-stimulation (Panel A) and for Nef at 60min post-stimulation (Panel B).
