# Supplementary material for: Mycobacterium tuberculosis and Human Immunodeficiency Virus Type 1 Cooperatively Modulate Macrophage Apoptosis via Toll Like Receptor 2 and Calcium Homeostasis
Source: PLoS One. 2015 Jul 1;10(7):e0131767. doi: 10.1371/journal.pone.0131767 (PMC4489497; doi:10.1371/journal.pone.0131767)
Supplement: S3 Fig — PMA stimulated THP1 cells were stimulated with 20 μg/ml Rv3416 or 15 μg/ml Nef or both. After 48 h cytoplasmic and mitochondrial extracts were prepared and western blotted for Cytochrome C levels. One of two independent experiments is shown. (DOCX) [file pone.0131767.s003.docx]

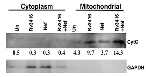


**S3 Fig. Cytochrome C is localized in mitochondria.** PMA stimulated THP1 cells were stimulated with 20 μg/ml Rv3416 or 15 μg/ml Nef or both. After 48 h cytoplasmic and mitochondrial extracts were prepared and western blotted for Cytochrome C levels. One of two independent experiments is shown.
