# Supplementary material for: Mycobacterium tuberculosis and Human Immunodeficiency Virus Type 1 Cooperatively Modulate Macrophage Apoptosis via Toll Like Receptor 2 and Calcium Homeostasis
Source: PLoS One. 2015 Jul 1;10(7):e0131767. doi: 10.1371/journal.pone.0131767 (PMC4489497; doi:10.1371/journal.pone.0131767)
Supplement: S4 Fig — PMA stimulated THP1 cells were stimulated with either 20 μg/ml Rv3416 or 15μg/ml Nef or both for 24h. Panel A, Cells were stained with Annexin V-APC and propidium Iodide (PI) and analyzed by flow cytometry. The percentage of cells positive for PI or/and Annexin V-APC are indicated inside the quadrants. Data from one of three independent experiments are shown. Panel B, shows percentage cell viability as determined by MTT assay. Unstimulated cells were taken as 100% viable and cell viability was calculated as percentage of unstimulated cell. Each experiment was performed in triplicate (n = 3). For Panel C, cells were stimulated with 20 μg/ml Rv3416 or 15μg/ml Nef or both and incubated for 24h. Cells were further incubated with 2 μM JC-1 dye for 30 min and analyzed by confocal microscopy. Green color indicate the loss of mitochondrial membrane potential while red color indicate the intact mitochondrial membrane potential. (DOCX) [file pone.0131767.s004.docx]

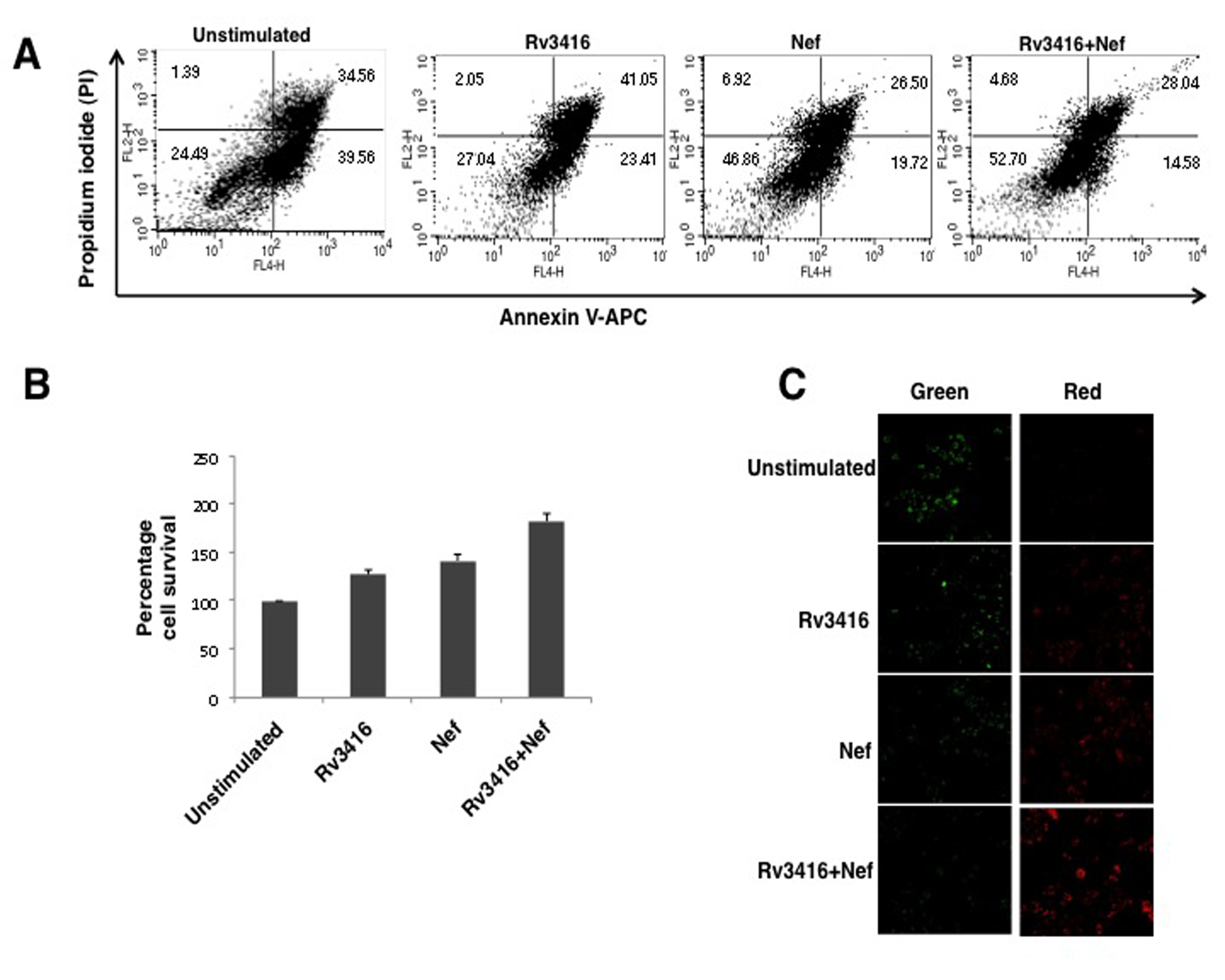


**S4 Fig. Rv3416 and Nef modulate apoptosis and not necrosis of macrophages.** PMA stimulated THP1 cells were stimulated with either 20 μg/ml Rv3416 or 15μg/ml Nef or both for 24h. Panel A, Cells were stained with Annexin V-APC and propidium Iodide (PI) and analyzed by flow cytometry. The percentage of cells positive for PI or/and Annexin V-APC are indicated inside the quadrants. Data from one of three independent experiments are shown. Panel B, shows percentage cell viability as determined by MTT assay. Unstimulated cells were taken as 100% viable and cell viability was calculated as percentage of unstimulated cell. Each experiment was performed in triplicate (n=3). Panel C, cells were stimulated with 20 μg/ml Rv3416 or 15μg/ml Nef or both and incubated for 24h. Cells were further incubated with 2 μM JC-1 dye for 30 min and analyzed by confocal microscopy. Green color indicate the loss of mitochondrial membrane potential while red color indicate the intact mitochondrial membrane potential.
