# Supplementary material for: Mycobacterium tuberculosis and Human Immunodeficiency Virus Type 1 Cooperatively Modulate Macrophage Apoptosis via Toll Like Receptor 2 and Calcium Homeostasis
Source: PLoS One. 2015 Jul 1;10(7):e0131767. doi: 10.1371/journal.pone.0131767 (PMC4489497; doi:10.1371/journal.pone.0131767)
Supplement: S5 Fig — PMA stimulated THP1 cells were stimulated with 1 μg/ml TLR2 ligand Pam3CSK4 for 24h and stained with Annexin V-APC. Thin line represents unstimulated cells while the dotted line represents stimulation with Pam3CSK4. (DOCX) [file pone.0131767.s005.docx]

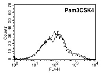


**S5 Fig. Stimulation of macrophages with TLR2 ligand Pam3CSK4 does not modulate apoptosis.** PMA stimulated THP1 cells were stimulated with 1 μg/ml TLR2 ligand Pam3CSK4 for 24h and stained with Annexin V-APC. Thin line represents unstimulated cells while the dotted line represents stimulation with Pam3CSK4.
