# Supplementary material for: Mycobacterium tuberculosis and Human Immunodeficiency Virus Type 1 Cooperatively Modulate Macrophage Apoptosis via Toll Like Receptor 2 and Calcium Homeostasis
Source: PLoS One. 2015 Jul 1;10(7):e0131767. doi: 10.1371/journal.pone.0131767 (PMC4489497; doi:10.1371/journal.pone.0131767)
Supplement: S7 Fig — Peripheral blood mononuclear cells were enriched from whole blood and monocytes were differentiated into macrophage as described in Materials and methods. Macrophages were stimulated with stimulated with 1 μg/ml TLR2 ligand Pam3CSK4 (Pam) and either 20 μg/ml Rv3416 or 15μg/ml Nef or both for 24h and stained with Annexin V-APC and analyzed by flow cytometry. Thin line represents the cells stimulated with 1 μg/ml TLR2 ligand Pam3CSK4 (Pam). Thick line represents cells stimulated with as indicated in the histogram. Data from one of three independent experiments are shown. (DOCX) [file pone.0131767.s007.docx]

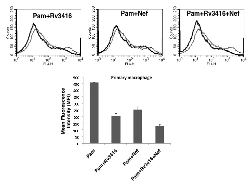


**S7 Fig. Rv3416 and Nef synergistically inhibit blood monocyte derived macrophages.** Peripheral blood mononuclear cells were enriched from whole blood and monocytes were differentiated into macrophage as described in Materials and methods. Macrophages were stimulated with stimulated with 1 μg/ml TLR2 ligand Pam3CSK4 and either 20 μg/ml Rv3416 or 15μg/ml Nef or both for 24h and stained with Annexin V-APC and analyzed by flow cytometry. Thin line represents the cells stimulated with 1 μg/ml TLR2 ligand Pam3CSK4 (Pam). Thick line represents cells stimulated with as indicated in the histogram. Data from one of three independent experiments are shown.
