# Supplementary material for: Mycobacterium tuberculosis and Human Immunodeficiency Virus Type 1 Cooperatively Modulate Macrophage Apoptosis via Toll Like Receptor 2 and Calcium Homeostasis
Source: PLoS One. 2015 Jul 1;10(7):e0131767. doi: 10.1371/journal.pone.0131767 (PMC4489497; doi:10.1371/journal.pone.0131767)
Supplement: S8 Fig — PMA stimulated THP1 cells were stimulated with 20 μg/ml Rv3416 or 15 μg/ml Nef or both for 24h along with ligands to TLR4 (0.1 μg/ml LPS) or TLR7 (1.0 μg/ml Imiquimod) or TLR9 (2.0 μg/ml CpG DNA) or DC-SIGN (0.5 μg/ml mannosylated Lipoarabinomanan; manLAM). For Panel A, cells were stained with Annexin V-APC. Thin lines represent stimulation with respective TLR ligands alone while the thick lines represent stimulation with respective TLR ligands with or without indicated antigens. For Panel B, cytoplasmic extracts from cells stimulated as above were western blotted for indicated molecules. Numbers below the blots indicate the relative intensities of the bands. (DOCX) [file pone.0131767.s008.docx]

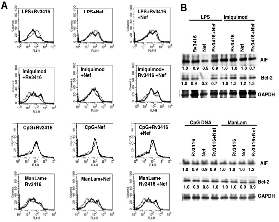


**S8 Fig. Inhibition of apoptosis by Rv3416 and Nef is not mediated via TLR4 or TLR7 or TLR9 or DC-SIGN.** PMA stimulated THP1 cells were stimulated with 20 μg/ml Rv3416 or 15 μg/ml Nef or both for 24h along with ligands to TLR4 (0.1 μg/ml LPS) or TLR7 (1.0 μg/ml Imiquimod) or TLR9 (2.0 μg/ml CpG DNA) or DC-SIGN (0.5 μg/ml mannosylated Lipoarabinomanan; manLAM). For Panel A, cells were stained with Annexin V-APC. Thin lines represent stimulation with respective TLR ligands alone while the thick lines represent stimulation with respective TLR ligands with or without indicated antigens. For Panel B, cytoplasmic extracts from cells stimulated as above were western blotted for indicated molecules. Numbers below the blots indicate the relative intensities of the bands.
