# Supplementary material for: Mycobacterium tuberculosis and Human Immunodeficiency Virus Type 1 Cooperatively Modulate Macrophage Apoptosis via Toll Like Receptor 2 and Calcium Homeostasis
Source: PLoS One. 2015 Jul 1;10(7):e0131767. doi: 10.1371/journal.pone.0131767 (PMC4489497; doi:10.1371/journal.pone.0131767)
Supplement: S9 Fig — PMA stimulated THP1 cells were stimulated with 1 μg/ml TLR2 ligand Pam3CSK4 and along with 20 μg/ml Rv3416 and 15μg/ml Nef or EGTA or TMB-8 for 24h. Panel A, Cells were stained with Annexin V-APC and Propidium Iodide (PI) and analyzed by flow cytometry. The percentage of cells positive for PI or/and Annexin V-APC are indicated inside the quadrants. Data from one of three independent experiments are shown. Panel B, shows percentage cell viability as determined by MTT assay. Pam+Rv3416+Nef stimulated cells were taken as 100% viable and cell viability was calculated as percentage of Pam+Rv3416+Nef stimulated cell. Each experiment was performed in triplicate (n = 3). (DOCX) [file pone.0131767.s009.docx]

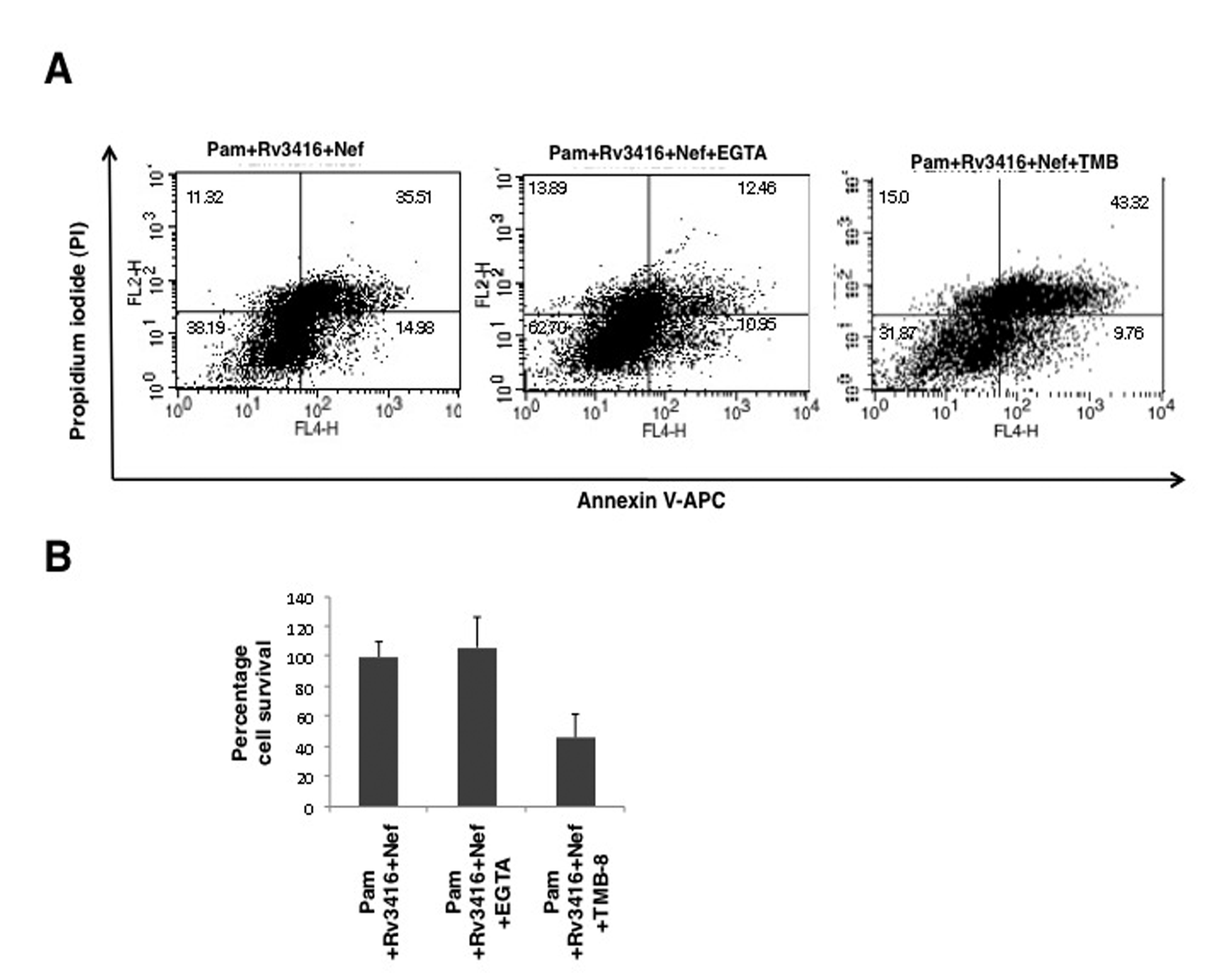


**S9 Fig. Calcium homeostasis differentially modulates Rv3416 and Nef induced apoptosis of macrophages.** PMA stimulated THP1 cells were stimulated with 1 μg/ml TLR2 ligand Pam3CSK4 and along with 20 μg/ml Rv3416 and 15μg/ml Nef or EGTA or TMB-8 for 24h. Panel A, Cells were stained with Annexin V-APC and Propidium Iodide (PI) and analyzed by flow cytometry. The percentage of cells positive for PI or/and Annexin V-APC are indicated inside the quadrants. Data from one of three independent experiments are shown. Panel B, shows percentage cell viability as determined by MTT assay. Pam+Rv3416+Nef stimulated cells were taken as 100% viable and cell viability was calculated as percentage of Pam+Rv3416+Nef stimulated cell. Each experiment was performed in triplicate (n=3).
