# Supplementary material for: Mycobacterium tuberculosis and Human Immunodeficiency Virus Type 1 Cooperatively Modulate Macrophage Apoptosis via Toll Like Receptor 2 and Calcium Homeostasis
Source: PLoS One. 2015 Jul 1;10(7):e0131767. doi: 10.1371/journal.pone.0131767 (PMC4489497; doi:10.1371/journal.pone.0131767)
Supplement: S10 Fig — PMA stimulated THP1 cells were treated with 50 μM L-NAME for 1h followed by stimulations with 1 μg/ml Pam3CSK4 along with 20 μg/ml Rv3416 and 15 μg/ml Nef for 24h. For Panel A, cells were stained with Annexin V-APC. Thin line represent cells stimulated in the absence of H2O2 while the thick line represent cells stimulated in the presence of L-NAME. Data from one of three independent experiments are shown. For Panel B, PMA stimulated cells were stimulated as indicated for 24h and cytoplasmic extracts were probed for indicated molecules and analyzed by western blots. Numbers below the blots indicate the relative intensities of the bands. Data from one of three experiments are shown. (DOCX) [file pone.0131767.s010.docx]

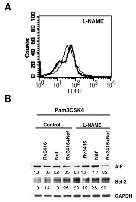
­­

**S10 Fig. Apoptosis in macrophages by Rv3416 and Nef does not involve inducible nitric oxide synthase.** PMA stimulated THP1 cells were treated with 50 μM L-NAME for 1h followed by stimulations with 1 μg/ml Pam3CSK4 along with 20 μg/ml Rv3416 and 15 μg/ml Nef for 24h. For Panel A, cells were stained with Annexin V-APC. Thin line represent cells stimulated in the absence of H_2_O_2_ while the thick line represent cells stimulated in the presence of L-NAME. Data from one of three independent experiments are shown. For Panel B, PMA stimulated cells were stimulated as indicated for 24h and cytoplasmic extracts were probed for indicated molecules and analyzed by western blots. Numbers below the blots indicate the relative intensities of the bands. Data from one of three experiments are shown.
